# A review of brain stimulation and neuromodulation therapies as a treatment of depression as a behavioural and psychological symptom of vascular dementia

**DOI:** 10.1192/j.eurpsy.2022.768

**Published:** 2022-09-01

**Authors:** T. Tiwary, D. John, S. De Souza

**Affiliations:** 1 University of Bristol, Bristol Medical School, Bristol, United Kingdom; 2 Somerset NHS Foundation Trust, Mental Health And Learning Disability, Taunton, United Kingdom

**Keywords:** vascular dementia, Depression, ECT, brain stimulation therapies

## Abstract

**Introduction:**

Vascular dementia (VaD) accounts for approximately 15% of all cases of dementia. While there are many different definitions of vascular dementia, it is generally understood to refer to “disease with a cognitive impairment resulting from cerebrovascular disease and ischaemic or haemorrhagic brain injury”. Research suggests that 30% of patients with VaD also suffer from depression. The treatment of depression in VaD with pharmacological therapy is relatively well-established, with the first line drug being a selective serotonin reuptake inhibitor (SSRI). However, a relatively under-researched area is the use of brain stimulation and neuromodulation therapies for the treatment of depression in VaD.

**Objectives:**

This review aims to provide a critical analysis on the efficacy and safety of brain stimulation therapies in treating depression in VaD to determine whether it is an appropriate treatment option.

**Methods:**

The databases used were PubMed and WebofScience. The available literature was analysed which resulted in three papers which met the inclusion criteria and were critically appraised.

**Results:**

In all three studies, depressive symptoms improved after ECT was administered, regardless of the specific tool used to measure the severity of depression. The side effects experienced were also only temporary and resolved independently which speaks to the safety of ECT as a treatment option.

**Conclusions:**

The results of the study prove that ECT is a safe and effective option in treating depression in VaD. However, more research is needed for the medical community to fully understand the different treatment options and say with certainty which is the safest and most effective.

**Disclosure:**

No significant relationships.

